# Effect of Culturally Mediated Right-Favoritism on the Direction of Pseudoneglect on Line Bisection Tasks

**DOI:** 10.3389/fpsyg.2021.756492

**Published:** 2021-10-22

**Authors:** Taim A. Muayqil, Ghadah M. Alhaidari, Lamia A. Alkuwaiz, Nouf A. Alotaibi, Hadeel K. Awartani, Alanoud A. Almufarrej, Ghadah S. Alqarni, Walid Alkeridy, Mohammed H. Alanazy

**Affiliations:** ^1^Neurology Unit, Department of Medicine, College of Medicine, King Saud University, Riyadh, Saudi Arabia; ^2^College of Medicine, King Saud University, Riyadh, Saudi Arabia; ^3^Department of Medicine, College of Medicine, King Saud University, Riyadh, Saudi Arabia; ^4^Department of Medicine, Geriatric Division, University of British Columbia, Vancouver, BC, Canada

**Keywords:** Arabs, handedness, pseudoneglect, line bisection, cultural effects, language

## Abstract

**Objectives:** Arabs have a right-to-left language and engage in favoring of the right side or limb when implementing daily routine practices. The purpose of this research is to explore the effect this cultural attitude might have on pseudoneglect, by comparing with a southeast Asian sample that has a left-to-right language structure.

**Methods:** Participants were from two separate ethnic groups (Arabs and Filipinos), residing in Saudi Arabia, healthy individals 18 years and above were allowed to volunteer in the study. The participants were recruited at King Saud University Medical City and the general community by both convenience and snowball sampling. Social demographic information such as gender, age, years of education, dominant hand, was also documented. The line bisection task (LBT) contained 36 randomly assorted lines of three different lengths placed at five different locations on a white sheet. The percent deviation score (PDS) was used to quantify pseudo-neglect. Tests of statistical significance including *t*-tests and mixed-effects regression were performed to determine if differences existed among different demographic variables or among line properties, respectively.

**Results:** A total of 256 were enrolled (Arabs 52.3%). The overall PDS mean and standard deviation (SD) was −0.64 (2.87), *p* = 0.0004, which shows a significant leftward deviation in the entire cohort. PDS was −1.26 (2.68) in Filipinos, and −0.08 (2.94) in Arabs. The difference was statically significant (*p* < 0.0001). Mixed effects model showed positive changes in the PDS value as the length of the line increased (*p* < 0.0001) and as the line was more rightward placed (*p* < 0.0001). However, Filipino participants would still exhibit negative changes in the PDS value in comparison to Arabs (*p* < 0.0001); There were no significant associations between PDS and other factors such as age, years of education and gender.

**Conclusion:** Differences found here between two distinct ethnic groups support the hypothesis that certain cultural aspects such as language direction and other cultural practices influence direction and degree of pseudo-neglect.

## Introduction

Pseudoneglect, a physiological phenomenon first described in 1980 (Bowers and Heilman, 1980), is defined as a normal tendency of healthy individuals to shift spatial attention in a certain direction. The term is used to describe findings stating that normal individuals without any neurological problems would have a systematic asymmetry in spatial attention toward the left (Kisbourne, 1970; Jewell and McCourt, 2000). It mirrors the clinical condition hemispatial neglect displayed by patients with right parietal lobe damage that manifests as contralateral spatial attention disruption. This has an important implication for the interpretation of the directional deviations displayed by patients with neglect. A common tool used to investigate pseudoneglect and visuospatial attention is the line bisection test, which requires an individual to identify the exact middle of a line (Carone, 2007).

Converging evidence from a large body of literature has found that patients with neglect bisect the line far to the right of the center, whereas healthy subjects typically bisect lines with a minor left bias. This supports a right hemisphere bias for attention allocation and provides evidence that lateralized processes predominantly located in the right hemisphere are normally engaged in healthy subjects during tasks where patients with unilateral neglect failed. This proves the importance of the right frontoparietal network in attention allocation (Mennemeier et al., 1997).

In addition to the lateralized processing bias of the right hemisphere, handedness, gender, assigned hand use, and length and position of the line have all been identified as influencers on pseudoneglect direction during line bisection tasks (LBTs; Jewell and McCourt, 2000). Bias toward the left has been described to be larger in men (Jewell and McCourt, 2000), and multiple studies have demonstrated an attenuation generally of leftward biases with advancing age (Barrett and Craver-Lemley, 2008; Friedrich et al., 2018). Men may even exhibit a reduction in biases in either direction with increasing age (Barrett and Craver-Lemley, 2008; Chen et al., 2011; Friedrich et al., 2018). The length of the bisected line may also be a factor; for example, younger individuals would bisect to the left of the line center the shorter the line was and to the right the longer the lines were (Pierce, 2000), a phenomenon described as a cross-over effect (Pierce, 2000; Friedrich et al., 2018). Other proposed factors include scanning habits, which stem from the reading direction of participants; these are thought to govern scanning strategies used during the task and as the final result of perceptual asymmetries (Manning et al., 1990; Abed, 1991). In support of this, previous studies have found consistent leftward bias in native left to right readers and central or rightward bias in right to left readers in various visuospatial tasks (Abed, 1991; Morikawa and McBeath, 1992; Fagard and Dahmen, 2003; Heath et al., 2005; Chokron et al., 2009; Friedrich and Elias, 2014), including those of line bisection (Chokron and Imbert, 1993; Chokron and De Agostini, 1995; Chokron et al., 1997).

Perhaps among the most important factors in line bisection outcomes is native reading habits. A study conducted on 120 normal right-handed Israeli and French subjects (Chokron and Imbert, 1993) revealed that Israeli subjects bisected the line to the right of the center, whereas French subjects bisected the line to the left of the center. This demonstrates that reading habits’ may influence bisection, with a rightward bisection for right-to-left readers and a leftward deviation for left-to-right readers. This suggests that scanning direction and other reading habits may play a role in space utilization. However, a study conducted with the aim of investigating how an imposed scanning direction could influence space perception among normal dextral patients and those with neglect with opposite reading habits suggested that these scanning-related effects are not specific to patients with neglect but determine the perceptual organization of normal subjects (Chokron et al., 1998). Moreover, according to a recent study that investigated the development of visuospatial attention in 159 typically developing children, left-handed children had a more significant leftward deviation compared with right-handed children (Ickx et al., 2017). These results again support that handedness may also play a role in deviation.

One of the few studies exploring LBT performance in an Arab population yielded novel results, namely, a different pseudoneglect direction than what was previously found in Western studies. A 2019 study (Muayqil et al., 2019) analyzed the performance of healthy Arab volunteers in the LBT and revealed a rightward bias and a tendency for male participants to deviate more strongly than female participants. This type of gender difference is similar to that described in previous Western studies, despite the opposite direction of deviation. Education is a variable that has not been studied in detail with regard to pseudoneglect and is worth exploring, given that studies that explore visuospatial abilities are influenced by education, such as cancelation tasks (Azouvi et al., 2006; Brucki and Nitrini, 2008). Line bisection has been found to correlate with education in a Brazilian study (Luvizutto et al., 2020) but has not been found to be significantly related in Arabs (Muayqil et al., 2019). Hence, if pseudoneglect to the left represents a default state, then a person with higher education, in Arabic for example, might demonstrate an alternate pseudoneglect direction. Line positioning on a page has also yielded conflicting results (Mennemeier et al., 1997; Ellis et al., 2006; Learmonth et al., 2018; Learmonth and Papadatou-Pastou, 2021).

In this study, we aimed to investigate whether differences could be demonstrated between two ethnic groups that differed in both language direction and cultural or religious favoring of the right on the direction of pseudoneglect during tasks of line bisection. Here, we hypothesized that Arabs would have a relatively more rightward position for line bisection in comparison with the southeast Asian group. The influence of ethnicity, education, age, gender, and line characteristics was also explored.

## Materials and Methods

### Participants

Participants were from two separate ethnic groups: Arabs residing in Saudi Arabia (predominantly Saudi Arabian) and Southeastern Asians that consisted entirely of Filipino nationals. In both groups, participants 18 and above were allowed to volunteer in the study. They were healthy individuals who were able to give consent. Those who suffered from disorders involving the central or peripheral nervous system, such as vascular disease, infectious diseases, trauma, disorders secondary to toxic or metabolic states, neurodegenerative disorders, autoimmune diseases, active systemic diseases, and malignancies, were excluded from the study. Furthermore, the exclusion criteria for Arabs included those who were exposed to any foreign language at an early age, living in a foreign country during early childhood, and those who were enrolled in international schools to eliminate any factors that could introduce novel reading or scanning strategies. For the Filipino population, being Muslim was an exclusion because of their exposure to Arabic and other Islamic teachings that encourage right-handedness. Those who could read or write in Arabic were also excluded. The participants were recruited by medical students at King Saud University Medical City and the general community by both convenience and snowball sampling. Both populations were divided into five age groups: 18–29, 30–39, 40–49, 50–59, and 60+ years, which were further divided by gender to ensure equal presentation of all the age groups and genders of both populations. Social demographic information, such as gender, age, years of education, and dominant hand, was also documented. Education was divided into two groups (grade 12 or less and >12th grade). Hand dominance was determined by self-identification.

### Procedures

The LBT contained 18 randomly assorted lines of three different lengths on a horizontal A4 paper. Each participant would complete two line-bisection sheets (A and B), where form B was an inverted version of form A. The tasks were conducted in well-lit quiet rooms to aid in concentration. The examiners would place the first paper horizontally in front of the participant and provide instructions to bisect each line in its center with a specified hand. Upon completion, the examiner would take the first sheet, place the second sheet, and the opposite hand would be used to complete it. The participants were pseudorandomized so that they were alternately assigned to complete either sheet A or sheet B first. They were instructed not to rotate the paper or erase or change their marks and to use their corrective eyewear if needed.

The lines were drawn and divided equally into three lengths (6, 12, and 18 cm). Each paper had 18 lines for a total of 36 lines. Each line was 3 mm thick, with a space of 1 cm between each line and the next. The first line was 1.5 cm from the upper edge of the paper, and the last line was 1 cm from the lower edge. The exact midpoints of the lines were measured and divided into three categories: 0 cm (the true center of the paper), 2.5 cm (from the true center), and 5 cm (from the true center). To ensure no recurring pattern, we used simple randomization to distribute the lines to five locations on the sheet: far-left, left, center, right, and far-right. This was also made to limit the ability of the participants to use a preceding line as a visual cue. The participants were asked to mark where they thought the center of the line was with either a pen or pencil. They would do this for each line and on both sheets. They were allowed to wear their corrective eye wear if needed and sit at a comfortable reading distance from the sheet of paper (30–45 cm). They were not allowed to move the sheet in any other orientation or raise it from the table. They were allowed to complete the task at their own pace but had to bisect lines from top to bottom.

The examiners would then measure the distance of the participants’ marks from the actual midpoint for every line in both forms. Any mark with a deviation of less than 1 mm was considered in the center. A frequently used percent deviation score (PDS; Scarisbrick et al., 1987; Hausmann et al., 2002; Facchin et al., 2016; Ickx et al., 2017) was used to quantify the amount of deviation by subtracting the actual left half of the line from the left half of the line, as marked by the participant, dividing it by the actual half of the line length, and then multiplying it by one hundred.

### Analysis

The mean PDS was calculated for each participant. Each participant’s PDS illustrated an average score acquired from the 36 lines they bisected. To calculate the overall PDS mean, we averaged the PDS of every line bisected by each participant (36 lines). Negative and positive PDS values suggest leftward and rightward deviations, respectively. Descriptive statistics (mean and SD) were used to describe the quantitative and categorical variables. A one sample *t*-test was used to determine the significance of the bias measured with the PDS in comparison to a hypothesis of no measurable bias (PDS = 0). Bivariate statistical analysis was carried out using Chi-square and Student’s *t*-test. ANOVA was used to explore for statistical differences between age groups. Mixed-effects regression was performed to determine the PDS change with repeated measures on participants with each line length and each line position as dependent variables. A *p*-value of <0.05 and 95% CI were used to report the statistical significance and precision of results. The data were analyzed using STATA version 15.

## Results

### Demographics

The total number of participants in the study was 256, 134 (52.3%) of whom were of Arab ethnicity, and 122 (47.66%) were Filipino. The ages of the participants ranged from 18 to 76 years; the mean age was 40.48 years (SD = 12.78 years). The largest percentage of the participants belonged to the 30–39 years age bracket (30.86%), whereas participants aged 60+ years made up only 9.77% of the total sample size. The total number of females in the study was 132 (51.56%), and that of males was 124 (48.44%). The mean (SD) of years of education was 14.5 (3.85) years, with a range of 0–27 years. Among the 256 participants, 4 identified as ambidextrous (3 Arab and 1 Filipino), 23 as left-handed (14 Arab and 9 Filipino), and 229 as right-handed (117 Arab and 112 Filipino). Other demographic data are shown in Table 1.

**TABLE 1:** Participant demographic variables and percent deviation scores (PDS).

	Arab		Filipino	
	*M* (SD)/*N* (%)	PDS, *M* (SD)	*M* (SD)/*N* (%)	PDS, *M* (SD)
**Gender**				
Male	69 (51.49%)	−0.093 (2.77)	55 (45.08%)	−1.46 (2.74)
Female	65 (48.51%)	−0.068 (3.15)	67 (54.92%)	−1.11 (2.65)
**Age (years)**	40.83 (14.32) (Range 18–76)		40.09 (10.8) (*Range* 23–65)	
**Age category (*N*)**				
18–29 (57)	35	−0.19 (2.44)	22	−1.28 (3.06)
30–39 (79)	33	−1.16 (2.46)	46	−0.94 (2.7)
40–49 (51)	25	0.23 (2.32)	26	−1.96 (2.42)
50–59 (44)	25	1.11 (3.05)	19	−0.98 (2.45)
60 + (25)	16	0.03 (4.66)	9	−1.53 (3)
**Education (years)**	15.74 (4.42) (Range 0–27)		13.19 (2.52) (Range 4–22)	
**Education category (*N*)**				
≤12	27	0.102 (3.92)	50	−1.20 (3.15)
>12	107	−0.13 (2.66)	72	−1.32 (2.33)

### Line Bisection

The overall PDS mean (SD) and median were −0.64 (2.87) and −0.56, respectively. These results indicate the presence of a statistically significant degree of leftward pseudoneglect [*t*(255) = −3.6, *p* = 0.0004]. No significant difference was found between the mean (SD) for the PDS between sheets A and B [−0.13 (2.2) and −0.49 (2.6), respectively; *t*(510) = 1.7; *p* = 0.09; *d* = −0.15]. The PDS mean (SD) was −0.08 (2.94) with a median of 0.21 for Arabs and −1.26 (2.68) with a median of −1.23 for Filipinos. The PDS for Arabs did not demonstrate a statistical significance for pseudoneglect [*t*(133) = −0.3, *p* = 0.75]. This was significant, however, in the Filipino group [*t*(121) = −5.23, *p* < 0.0001]. The difference between both groups was significant [*t*(254) = −3.37, *p* = 0.001, *d* = 0.42], indicating that Filipinos had a larger leftward pseudoneglect than Arabs. The PDS by ethnicity, age, years of education, and gender is demonstrated in Table 1. A negative PDS was observed in 147 participants: 67 Arabs and 83 Filipinos. A positive PDS was found in 109 participants, in which 70 were Arab and 39 were Filipino.

ANOVA showed that the mean PDS did not differ among the age categories [*F* = (4, 251) 1.43, *p* = 0.23]. No significant difference was found between the two education categories [*t*(254) = −0.358, *p* = 0.72, *d* = −0.05]. Analysis of the PDS mean by gender yielded [*t*(254) = *p* = 0.78, *d* = −0.04], also showing no statistical significance.

Further analysis was done between the two ethnicities comparing each respective gender. The results showed a statistically significant difference between Filipino males and Arab males (*t* = 2.75, *p* = 0.007, *d* = −0.5) and between Filipino females and Arab females (*t* = 2.069, *p* = 0.04, *d* = −0.4) (Table 1).

The PDS differed according to the line position and length. The mixed-effects regression model results are presented in Table 2. A significantly positive increase in average PDS was observed when the task was completed on a longer line and when the line center was located further to the right of the page. Among the variables that significantly affected the PDS according to both line length and position were ethnicity and being left-handed, both being significantly associated with more negative PDS values (Table 2).

**TABLE 2:** Mixed effects model results for the PDS according to “line” length and location.

	PDS by length			PDS by location				
	6 cm, *M* (SD)	12 cm, *M* (SD)	18 cm, *M* (SD)	Far left, *M* (SD)	Left, *M* (SD)	Center, *M* (SD)	Right, *M* (SD)	Far right, *M* (SD)
	−1.01 (3.6)	−0.66 (3.3)	−0.22 (3.2)	−1.39 (4.6)	−0.42 (3.5)	−1.02 (3.2)	−0.61 (3.9)	0.21 (4.2)

	**Coefficient (SE)**	***t*, *p*-value**	**95% CI**	**Coefficient (SE)**		***t*, *p*-value**	**95% CI**	

**Line**								
Length/location	0.4 (0.09)	4.26, <0.0001	0.22, 0.58	0.27 (0.06)		4.46, <0.0001	0.15, 0.38	
**Gender**								
Female	Reference			Reference				
Male	0.01 (0.35)	0.03, 0.98	−0.67, 0.69	−0.03 (0.35)		−0.07, 0.94	−0.71, 0.66	
**Age**								
18–29	Reference			Reference				
30–39	−0.17 (0.48)	−0.37 (0.71)	−1.11, 0.76	−0.12 (0.48)		−0.25, 0.81	−1.05, 0.82	
40–49	0 (0.52)	0.00, 1	−1.03, 1.03	0.02 (0.53)		0.03, 0.97	−1.01, 1.05	
50–59	0.84 (0.54)	1.55, 0.12	−0.22, 1.91	0.86 (0.55)		1.57, 0.12	−0.2, 1.93	
60**+**	−0.02 (0.67)	−0.03, 0.97	−1.33, 1.28	0.14 (0.67)		0.21, 0.83	−1.17, 1.45	
**Education**								
≤12	Reference			Reference				
>12	−0.08 (0.39)	−0.2, 0.84	−0.85, 0.69	−0.09 (0.39)		−0.23, 0.82	−0.86, 0.68	
**Ethnicity**								
Arab	Reference			Reference				
Filipino	−1.26 (0.36)	−3.55, <0.0001	−1.96, −0.56	−1.31 (0.36)		−3.67, <0.0001	−2.01, −0.61	
Handedness*								
Right	Reference			Reference				
Left	−2.11 (0.56)	−3.75 (<0.0001)	−3.21, −1	−1.96 (0.56)		−3.49, <0.0001	−3.07, −0.86	

The top row shows the mean (standard deviation) of the PDS obtained for each line in relation to its length and in relation to its position on the page. The coefficient for line indicates average increase in PDS value with each longer line or each rightward positioned line. *The four self-identified as ambidextrous were included under left handed group.

## Discussion

This study is among the few describing line bisection discrepancies between populations with distinct cultural backgrounds. One group consisted of Arab individuals brought up in rightward-favoring cultures with a right-to-left direction of written language. In comparison, the other group was composed of Filipino individuals who represented a left-to-right language population with a culture permissive in laterality and choice of handedness.

Using samples from both populations with different age groups and education levels revealed an overall pseudoneglect to the left of the true center of each line. This overall result is consistent with previous studies that suggest the presence of a slight left bias in healthy people with intact right hemispheres (Jewell and McCourt, 2000; Çiçek et al., 2009). Despite this, when looking at the PDS mean of each ethnic group separately, a clear difference in the extent of pseudoneglect was observed in each population. In our study, the PDS mean of Filipinos was found to be significantly larger to the left, whereas the Arabs gave a much smaller degree of deviation that was not significant. The significant leftward deviation of Filipinos is in agreement with what most Western studies have described about the characteristics of pseudoneglect, and an influence from factors, such as written language direction and a lack of cultural preferences toward one direction over the other, is likely. This theory is supported by Friedrich and Elias (2014), who reported that leftward bias is only found in left-to-right language readers on a greyscale task. Although our PDS mean for the Arab population sample did not show a strong rightward deviation, unlike a previous study that gave a PDS mean of +1.57 (SD 3.4) to the right (Muayqil et al., 2019), our results showing no significant leftward pseudoneglect are still consistent. This finding resembles those described in a previous study of native right-to-left readers who did not have a demonstrable pseudoneglect (Friedrich and Elias, 2014) when performing the gray scale task. Rightward deviation has also been seen in older studies, such as in Chokron and Imbert (1993), in which Hebrew and French readers were compared using a line bisection test that displayed a rightward bias in Hebrew readers. We can assume that the lack of pseudoneglect found here and the rightward deviation found in Middle Eastern participants, among other studies mentioned, is likely to be caused by the reading direction of their native language and Middle Eastern and Islamic cultural favoritism to the right. Whether this rightward bias could dampen the clinical assessment of unilateral spatial neglect in Arab patients with parietal lesions is unknown. This will require further detailed studies of lesioned patients of different backgrounds.

While increasing age has been noted as a factor that would induce rightward deviation (Jewell and McCourt, 2000) and younger participants would display a leftward bias (Jewell and McCourt, 2000; Failla et al., 2003), we found no significant association between age of participants and PDS in our study. This could be due to the larger number of younger participants in the study, in which 73% of the participants were under 50 years old. Although values showed that females deviated slightly less than males, the association with gender did not reach statistical significance in our study. This is not entirely inconsistent with previous reports of a modest relation (Jewell and McCourt, 2000) showing that males were more likely to deviate to the left than females. Interestingly, a recent study that examined a group of right-handed patients’ performance on line bisection tests within 24 h of an acute right hemispheric stroke demonstrated no difference in relation to gender when performing the tasks for unilateral spatial neglect (Kleinman et al., 2008). Even when analysis was performed between the same genders from both ethnic groups, statistical significance was found. Taking into account the lack of an association between gender and the PDS, one can infer that inherent traits within each ethnicity influence the PDS.

As expected, the vast majority of participants were right-handed. Previous literature has explored the role of handedness in LBT performance and found that left-handed individuals made larger leftward errors when using their dominant hand compared with right-handed individuals using their right hand (Scarisbrick et al., 1987; Jewell and McCourt, 2000; Ickx et al., 2017; Muayqil et al., 2019). Here, we found that those who identified as left-handed were more likely to have a negative PDS value; however, the relatively small number of left-handed individuals limited the ability to explore further effects of the dominant hand on the degree of pseudoneglect in this study. This was, however, controlled for by having participants take equal random turns using each hand.

The rightward placement of a line on the sheet was associated with a more positive PDS. This appears to be consistent with multiple previous reports on the effect of line location (Jewell and McCourt, 2000). Also consistent with this finding is an earlier study of Arabs that showed increasing leftward errors with more leftward positioned lines (Muayqil et al., 2019). The crossover effect, a phenomenon where more rightward displacements occur with shorter lines in normal individuals and vice versa in neglect patients, has been previously described in studies but with no specified line length of when these phenomena could occur (Monaghan and Shillcock, 1998; Rueckert et al., 2002; Nicholls et al., 2016). Although we found more rightward deviations with a longer line length in this study, earlier studies have been inconsistent, with previous meta-analytical studies describing an overall increase in the same direction of the original bias, more leftward biases, or more rightward biases with increasing line length (Jewell and McCourt, 2000; Friedrich et al., 2018; Learmonth and Papadatou-Pastou, 2021).

While years of education yielded no significance in our study, it is a factor that could be further explored in future research. A recent proposed influencer on bisection results was the level of urbanization (Linnell et al., 2014), which may actually induce a leftward bias. As Riyadh is considered an urban city, it may be beneficial to compare the performance of groups from urban areas to rural areas. Another factor worth exploring is the knowledge of two languages with opposite script directions, which has been proposed to cause an attenuation of bias (Kazandjian et al., 2010). Most Arabs of recent generations are taught English early in school or have learned it when taking graduate degrees, and the population has some level of proficiency in the language. A study comparing bilingual Arabic and English speakers to monolingual Arabic speakers would be of interest.

### Limitations

Although our study was conducted on a number of participants and balanced for sex and hand use through different age categories, the proportion of younger individuals seems to be larger. This may have hindered finding a more accurate representation of age and PDS mean associations, which will require future studies on a larger number of the older population. In addition, we controlled only for hand use and not handedness; further exploration with validated handedness scales is needed. Other considerations include the limited number of test items in the current study and the absence of a controlling factor to discriminate reading direction effects from overall rightward favoring habits. Lastly, cultural differences may lead to differences in emotional processing, which has been described as playing a role in line bisection (Vicario et al., 2021).

## Conclusion

In conclusion, the present study allowed us to contrast the degree and direction of pseudoneglect for two ethnically distinct groups. The analysis supports the proposed hypothesis that culturally acquired cognitive strategies within different ethnicities influence the direction and magnitude of pseudoneglect when performing LBTs in healthy individuals. This is likely inferred from language direction and the existence of a cultural favoritism to the right.

## Data Availability Statement

The raw data supporting the conclusions of this article will be made available by the authors, without undue reservation.

## Ethics Statement

The studies involving human participants were reviewed and approved by Internal Review Board, College of Medicine, King Saud University. The patients/participants provided their written informed consent to participate in this study.

## Author Contributions

TM was involved in conception, supervision of research process, and analysis and drafting of manuscript. GAh, LA, NA, HA, AA, and GAq were involved in participant assessment, data collections, data management, and drafting of manuscript. WA and MA were involved in revision of analytic process and manuscript. All authors contributed to the article and approved the submitted version.

## Conflict of Interest

The authors declare that the research was conducted in the absence of any commercial or financial relationships that could be construed as a potential conflict of interest.

## Publisher’s Note

All claims expressed in this article are solely those of the authors and do not necessarily represent those of their affiliated organizations, or those of the publisher, the editors and the reviewers. Any product that may be evaluated in this article, or claim that may be made by its manufacturer, is not guaranteed or endorsed by the publisher.
